# Supplementary figures and images for: Sparse Codes for Speech Predict Spectrotemporal Receptive Fields in the Inferior Colliculus
Source: PLoS Comput Biol. 2012 Jul 12;8(7):e1002594. doi: 10.1371/journal.pcbi.1002594 (PMC3395612; doi:10.1371/journal.pcbi.1002594)

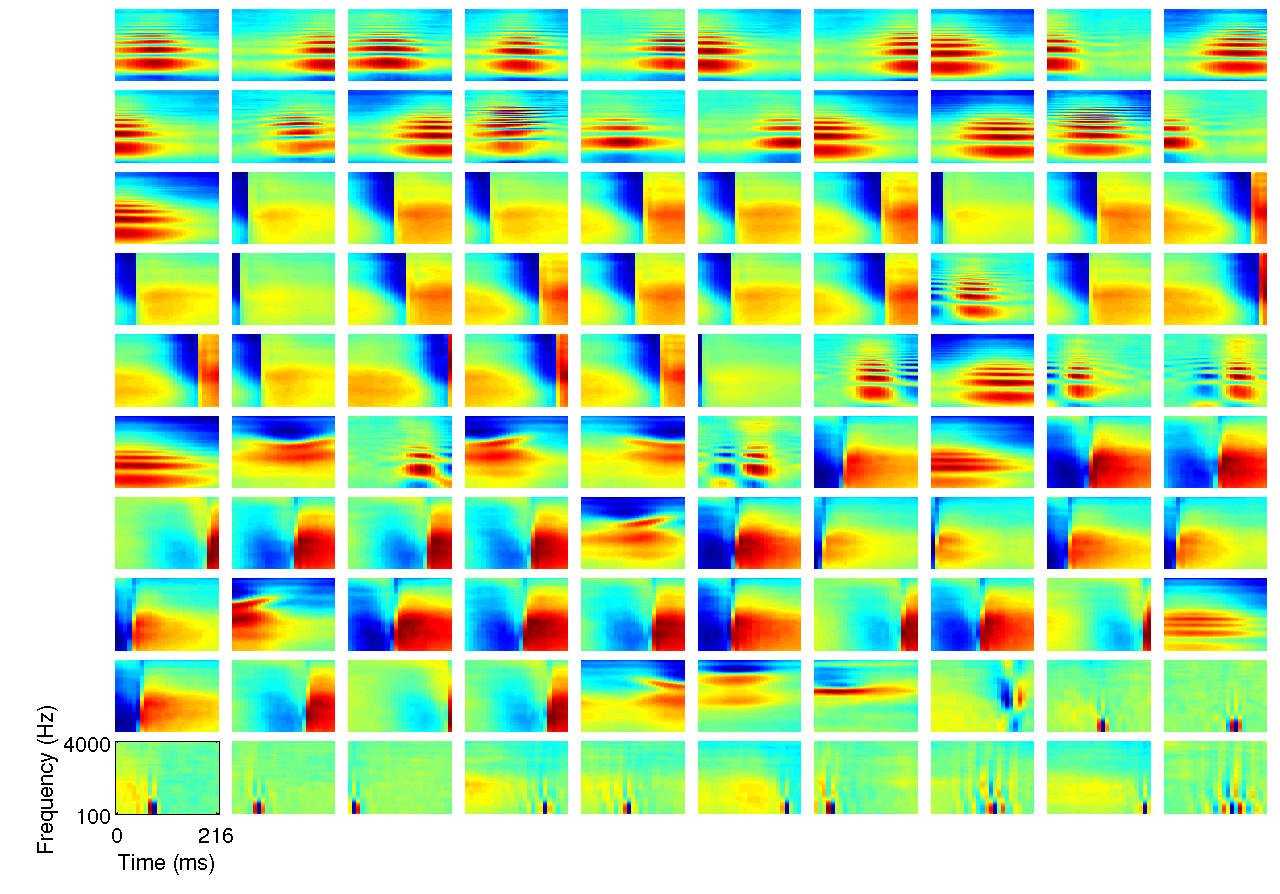

Supplement: Figure S1 — The full set of elements from a half-complete, L0-sparse dictionary trained with LCA [24] on spectrograms of speech. Each rectangle represents the spectrotemporal receptive field of a single element in the dictionary; time is plotted along the horizontal axis (from 0 to 216 msec), and log frequency is plotted along the vertical axis, with frequencies ranging from 100 Hz to 4000 Hz. Color indicates the amount of power present at each frequency at each moment in time, with warm colors representing high power and cool colors representing low power. Each element has been normalized to have unit Euclidean length. Elements are arranged in order of their usage during inference with usage increasing from left to right along each row, and all elements of lower rows used more than those of higher rows. (TIFF) [file pcbi.1002594.s001.tif]

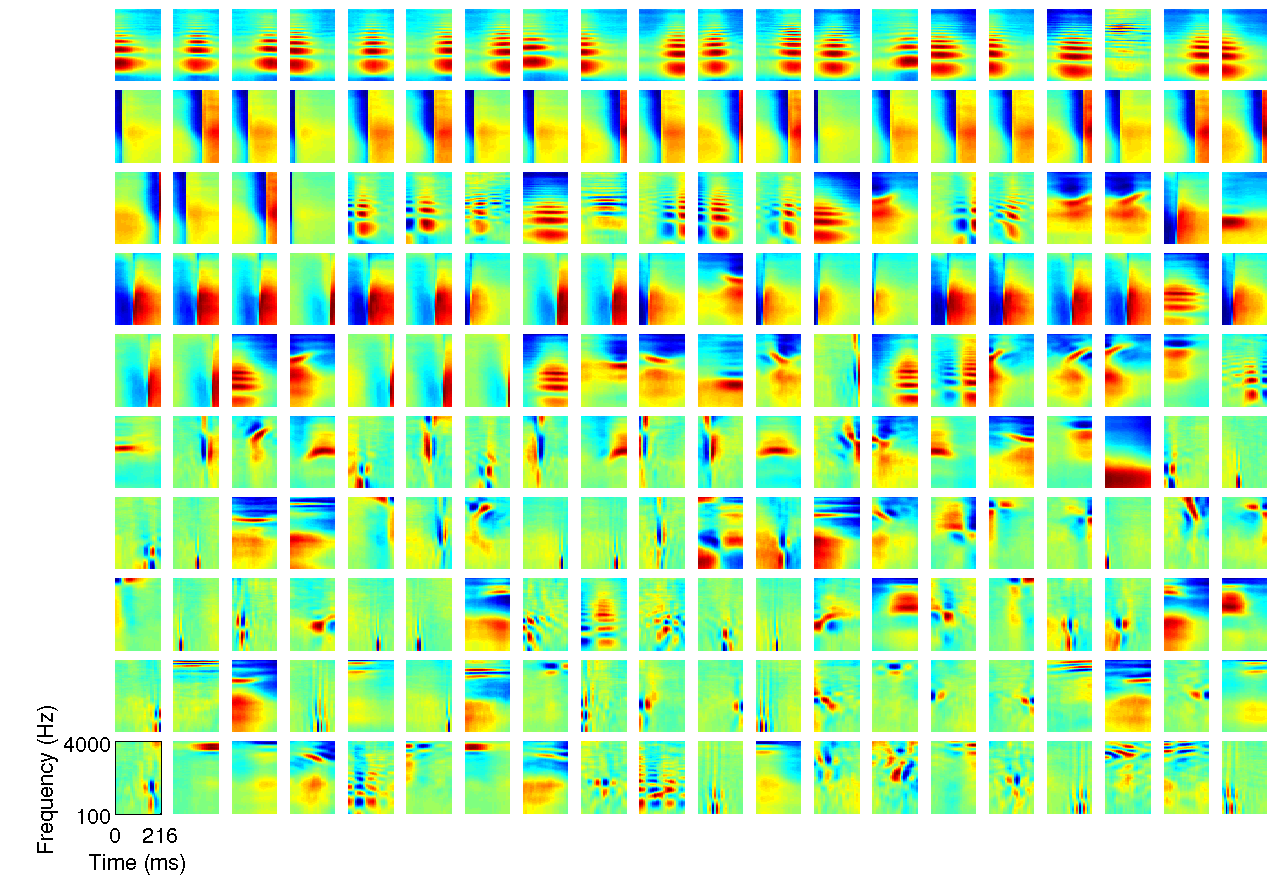

Supplement: Figure S2 — The full set of elements from a complete, L0-sparse dictionary trained with LCA [24] on spectrograms of speech. Same conventions as Fig. S1. (TIF) [file pcbi.1002594.s002.tif]

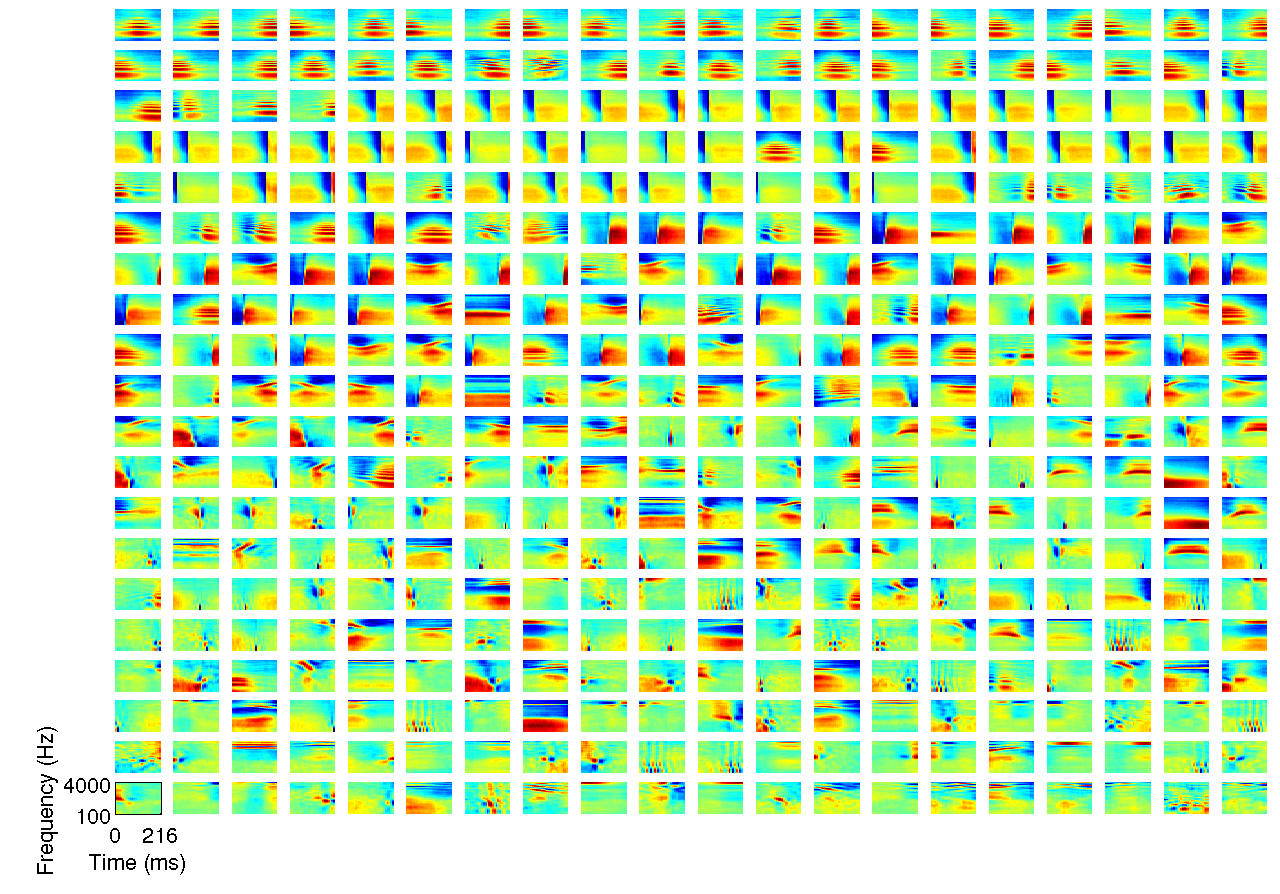

Supplement: Figure S3 — The full set of elements from a two times overcomplete, L0-sparse dictionary trained with LCA [24] on spectrograms of speech. Same conventions as Fig. S1. (TIF) [file pcbi.1002594.s003.tif]

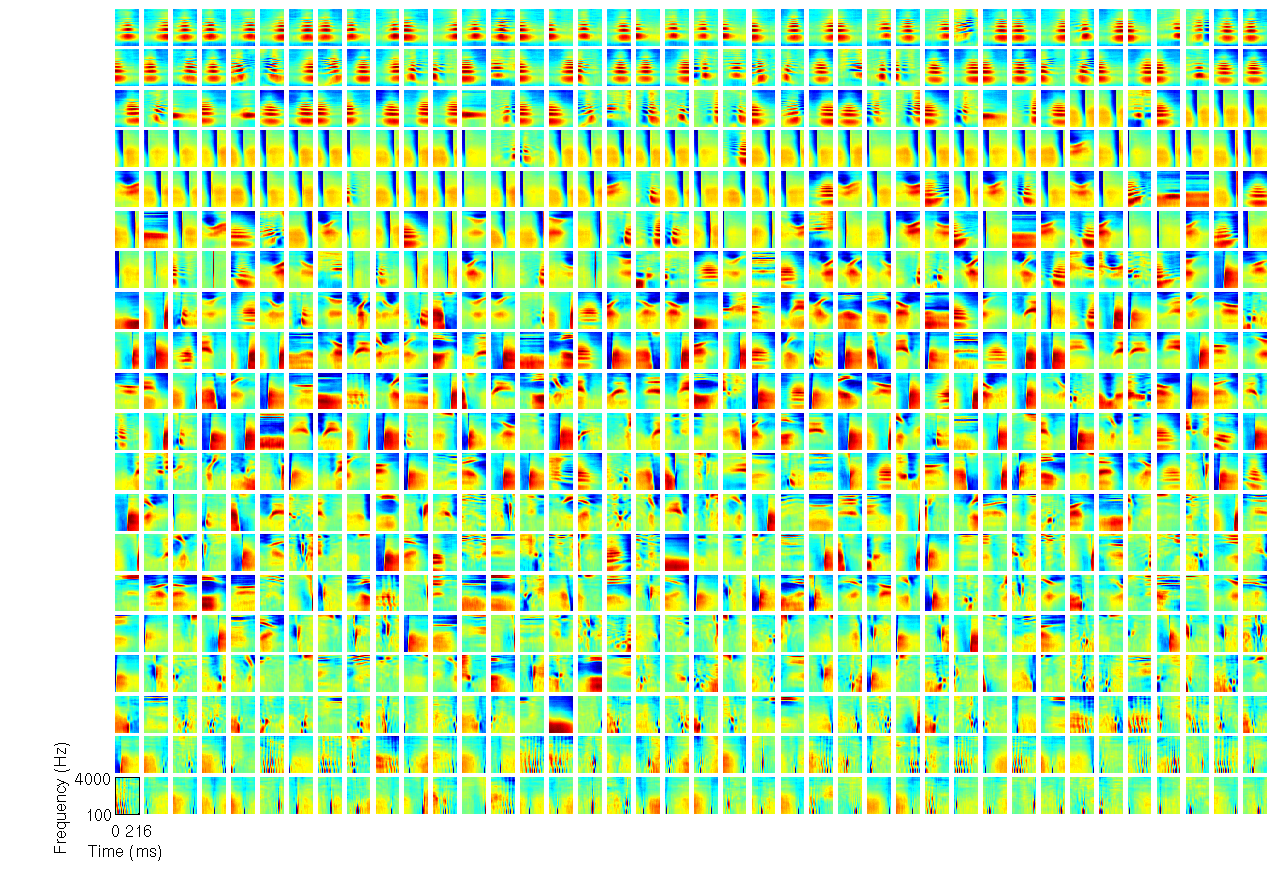

Supplement: Figure S4 — The full set of elements from a four times overcomplete, L0-sparse dictionary trained with LCA [24] on spectrograms of speech. Same conventions as Fig. S1. (TIF) [file pcbi.1002594.s004.tif]

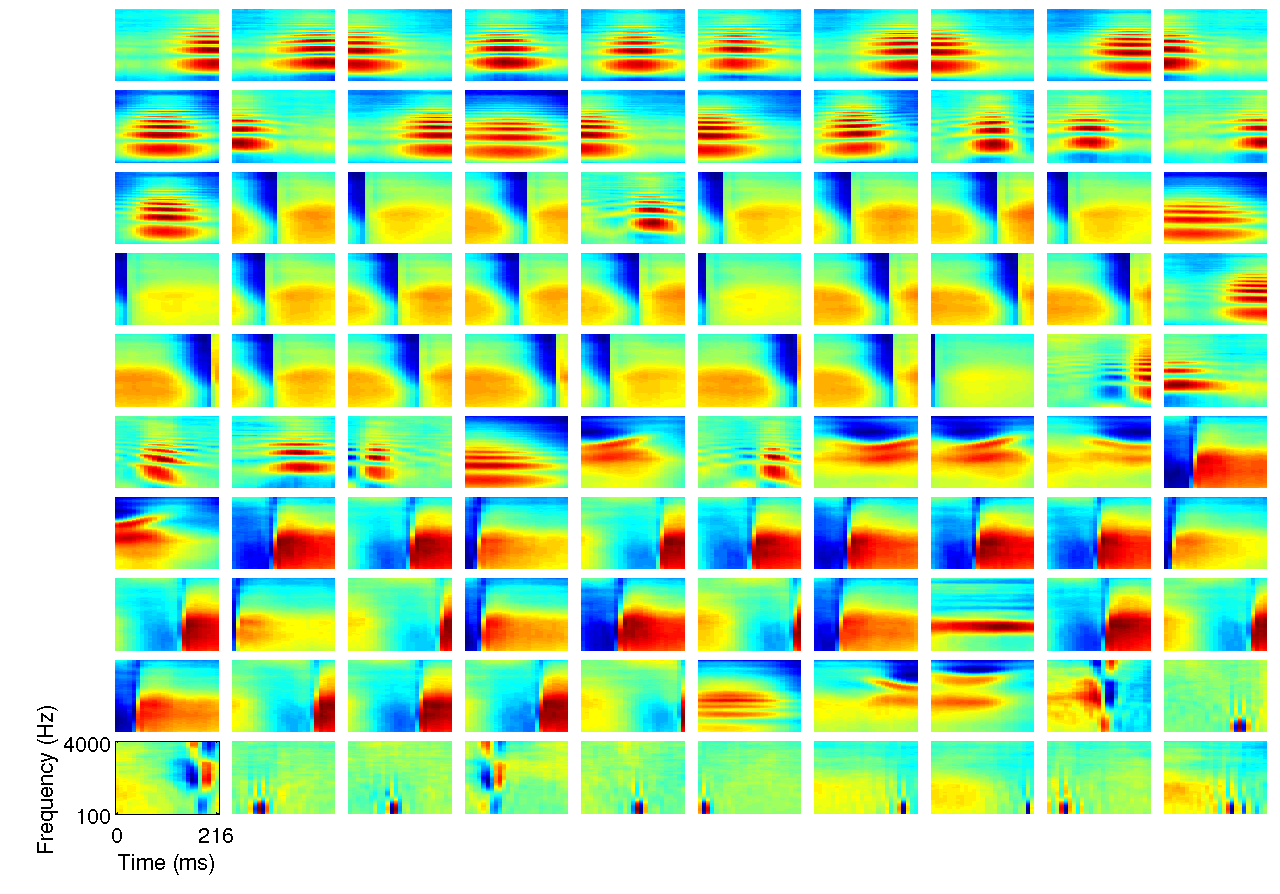

Supplement: Figure S5 — The full set of elements from a half-complete, L1-sparse dictionary trained with LCA [24] on spectrograms of speech. Same conventions as Fig. S1. (TIF) [file pcbi.1002594.s005.tif]

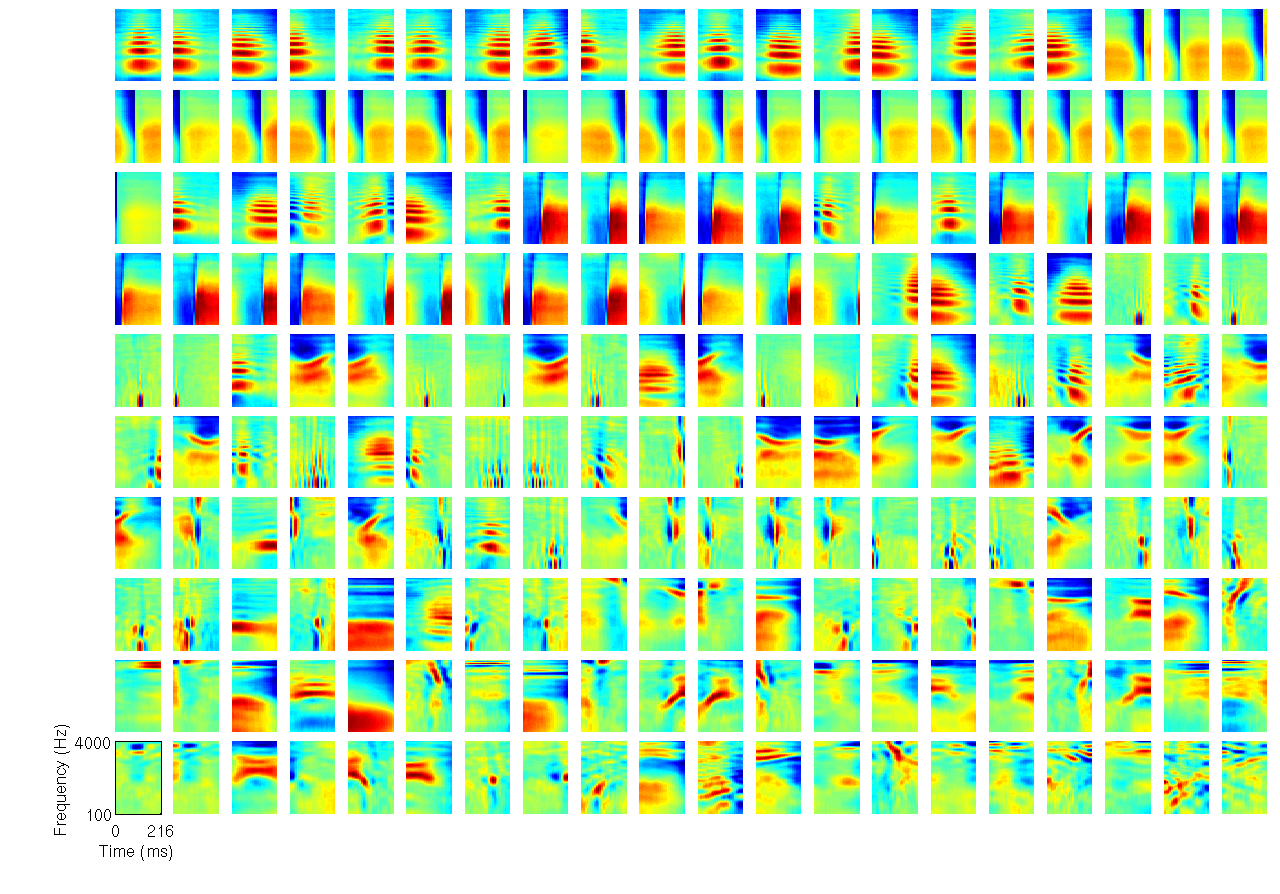

Supplement: Figures S6 — The full set of elements from a complete, L1-sparse dictionary trained with LCA [24] on spectrograms of speech. Same conventions as Fig. S1. (TIF) [file pcbi.1002594.s006.tif]

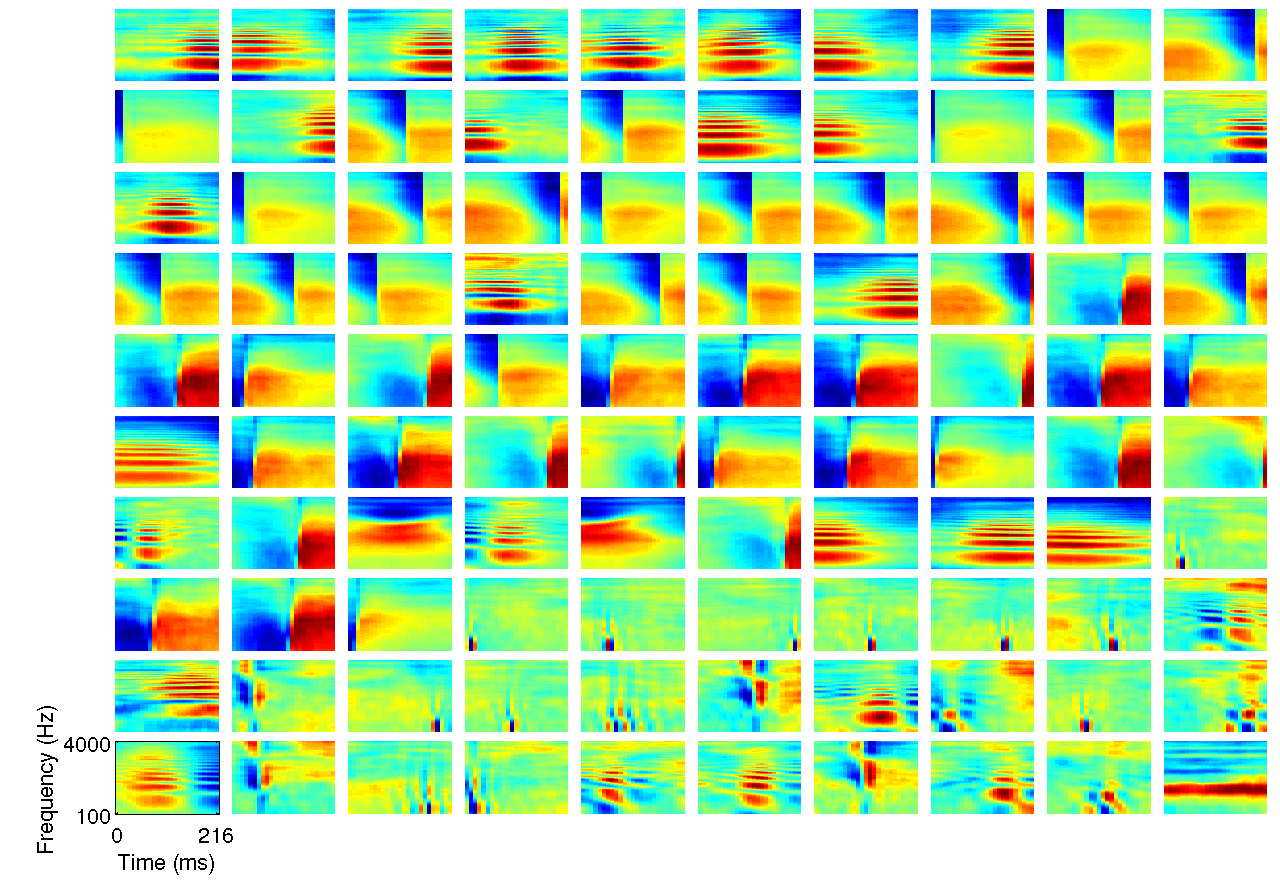

Supplement: Figure S9 — The full set of elements from a half-complete, L1-sparse dictionary trained with Sparsenet [10] on spectrograms of speech. Same conventions as Fig. S1. (TIF) [file pcbi.1002594.s009.tif]

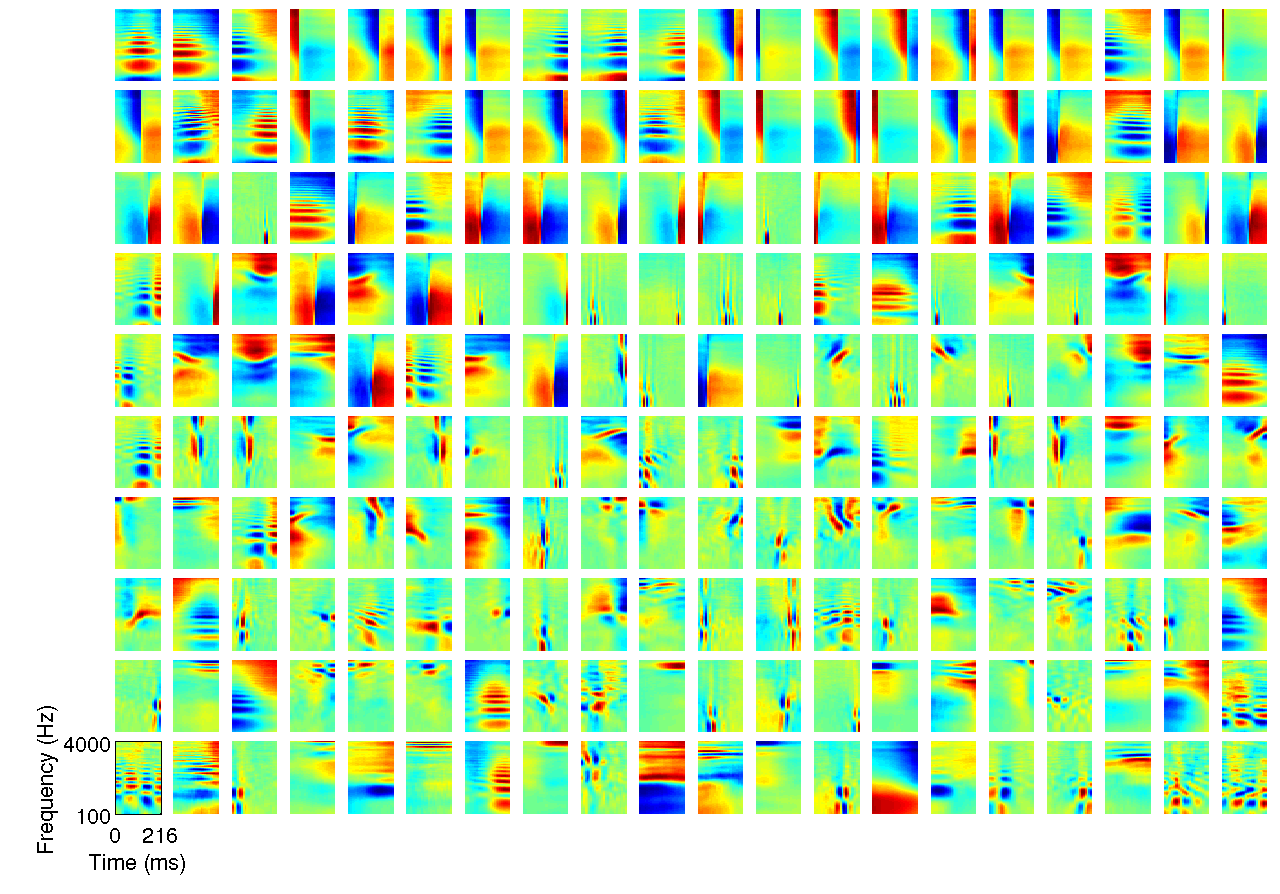

Supplement: Figure S10 — The full set of elements from a complete, L1-sparse dictionary trained with Sparsenet [10] on spectrograms of speech. Same conventions as Fig. S1. (TIF) [file pcbi.1002594.s010.tif]

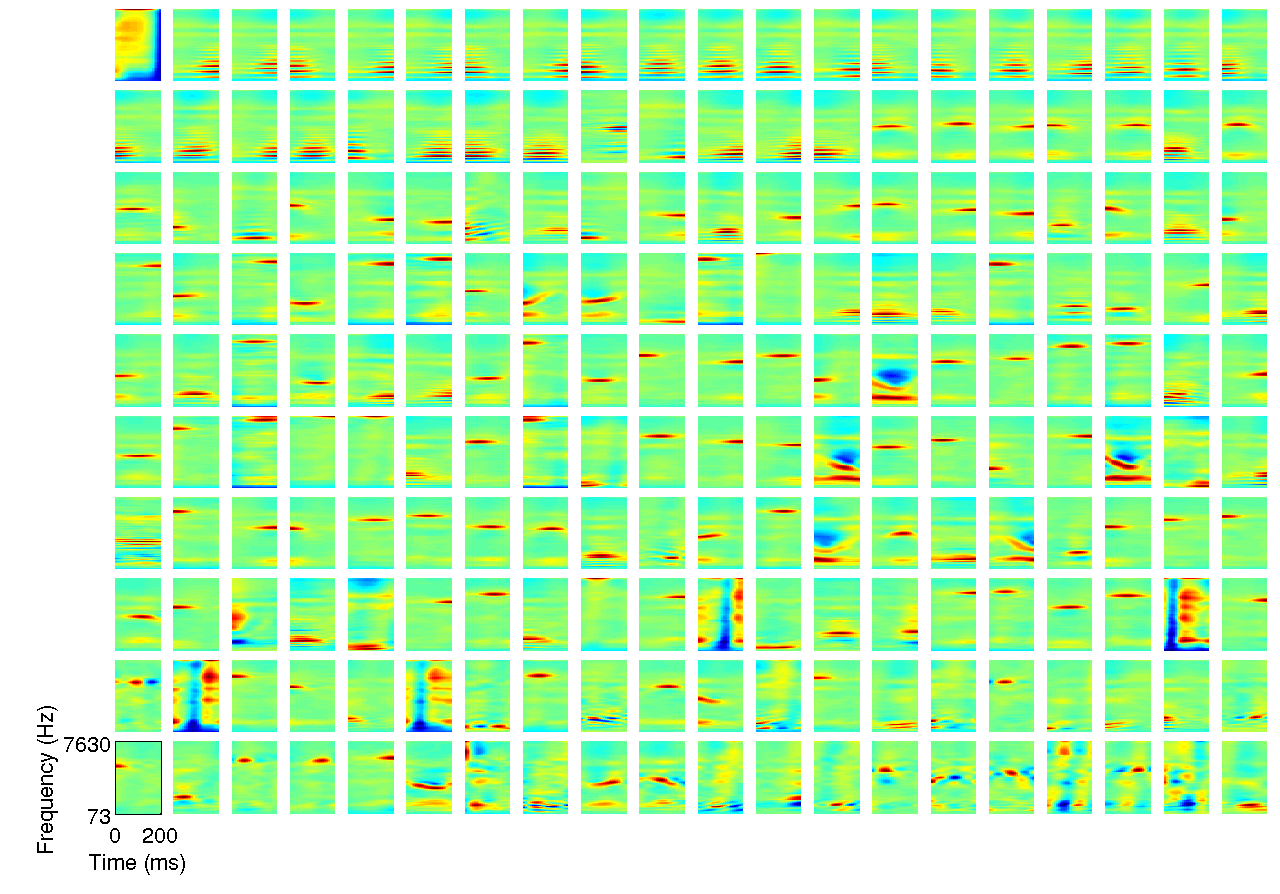

Supplement: Figure S11 — The full set of elements from a complete, L0-sparse dictionary trained using LCA [24] on cochleograms of speech. Each rectangle represents the spectrotemporal receptive field of a single element in the dictionary; time is plotted along the horizontal axis (from 0 to 250 ms), and log frequency is plotted along the vertical axis, with frequencies ranging from 73 Hz to 7630 Hz. Color indicates the amount of power present at each frequency at each moment in time, with warm colors representing high power and cool colors representing low power. Each element has been normalized to have unit Euclidean length. Elements are arranged in order of their usage during inference with usage increasing from left to right along each row, and all elements of lower rows used more than those of higher rows. (TIF) [file pcbi.1002594.s011.tif]

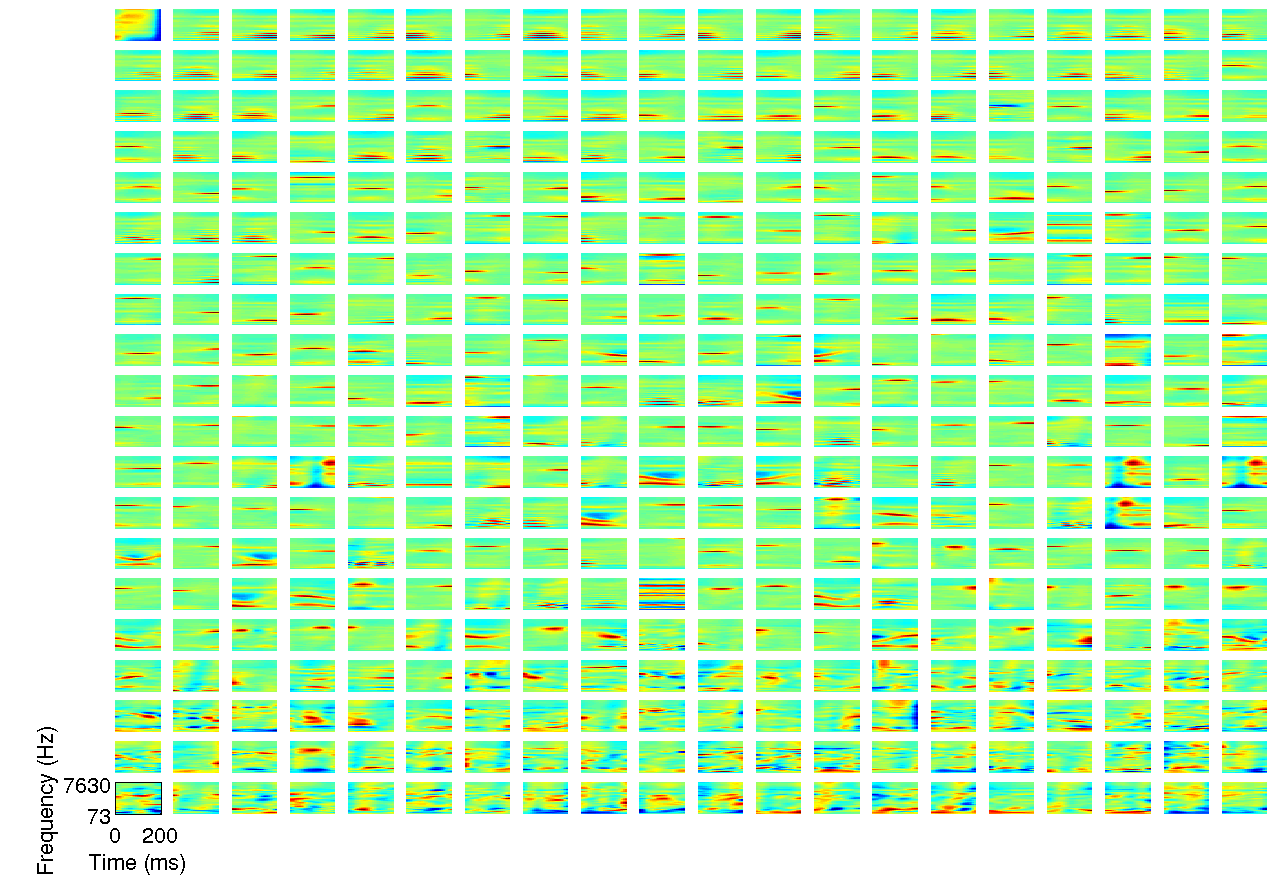

Supplement: Figure S12 — The full set of elements from a two times overcomplete, L0-sparse dictionary trained with LCA [24] on cochleograms of speech. Same conventions as Fig. S11. (TIF) [file pcbi.1002594.s012.tif]

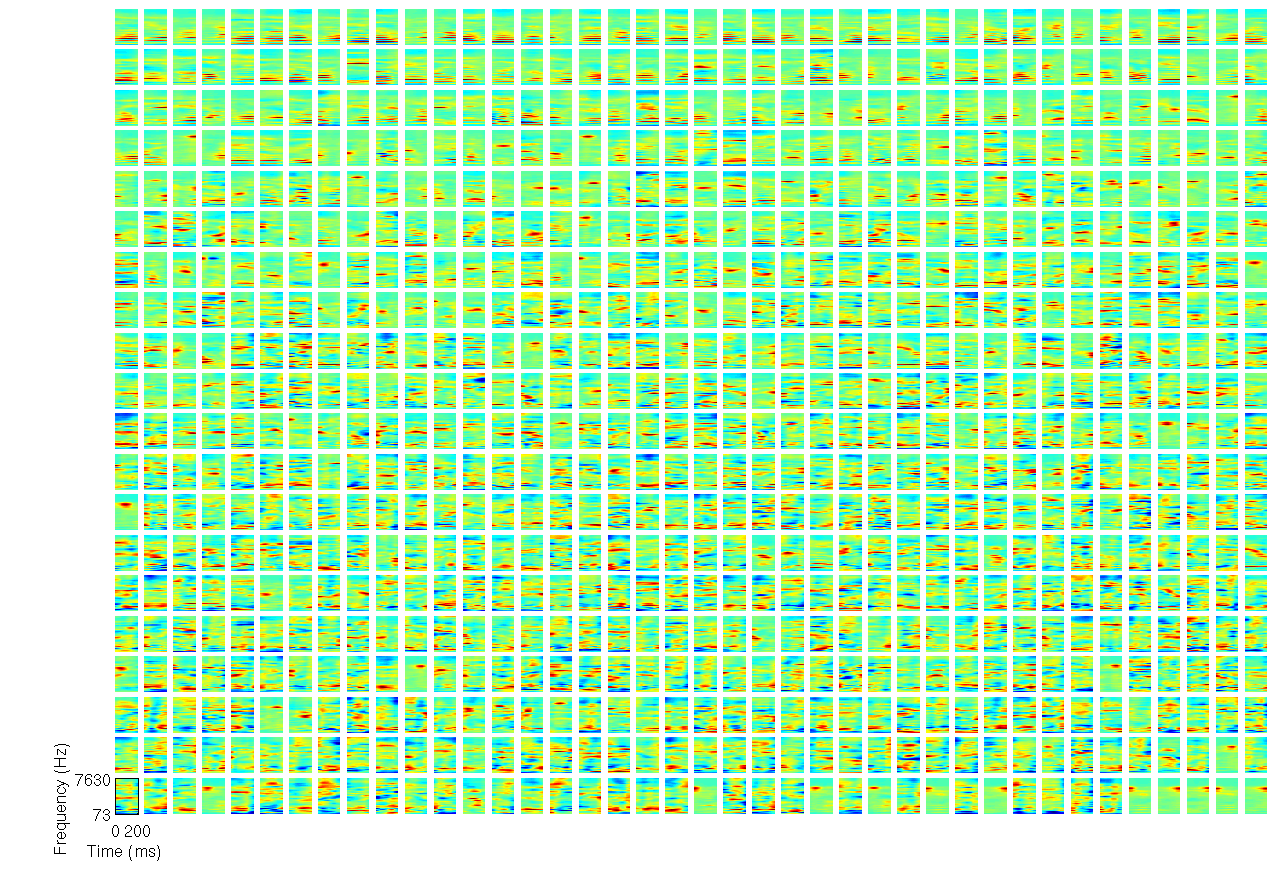

Supplement: Figure S13 — The full set of elements from a four times overcomplete, L0-sparse dictionary trained with LCA [24] on cochleograms of speech. Same conventions as Fig. S11. (TIF) [file pcbi.1002594.s013.tif]

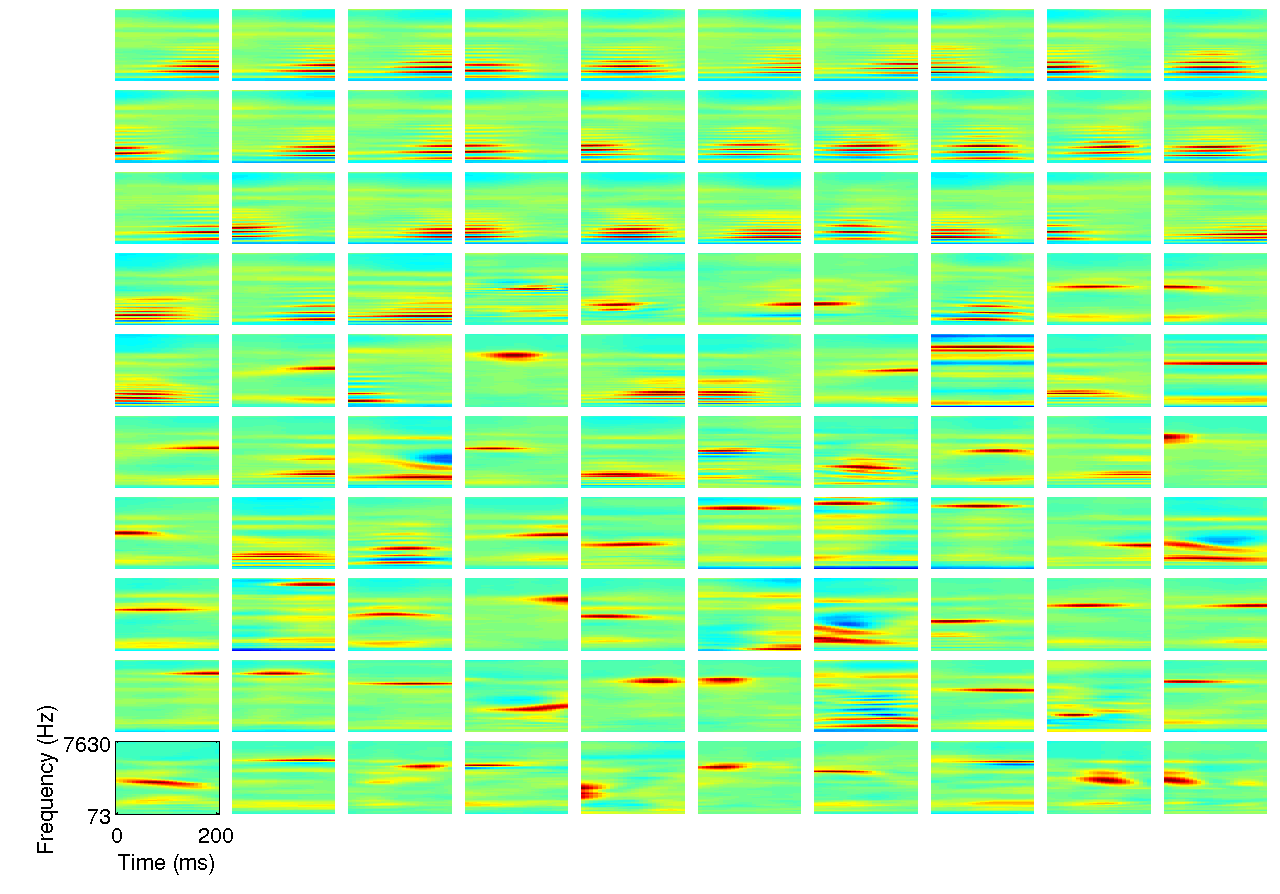

Supplement: Figure S14 — The full set of elements from a half-complete, L1-sparse dictionary trained with LCA [24] on cochleograms of speech. Same conventions as Fig. S11. (TIF) [file pcbi.1002594.s014.tif]

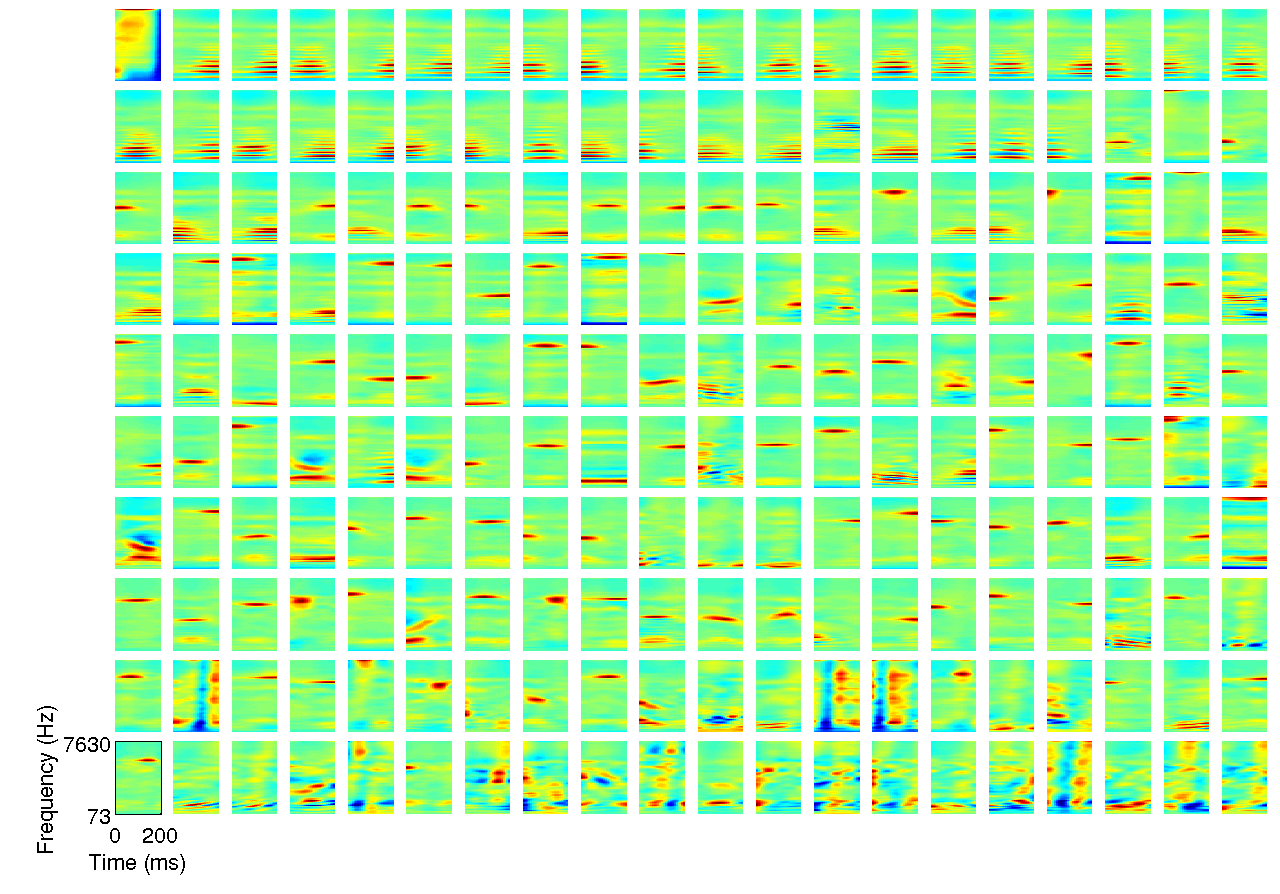

Supplement: Figure S15 — The full set of elements from a complete, L1-sparse dictionary trained with LCA [24] on cochleograms of speech. Same conventions as Fig. S11. (TIF) [file pcbi.1002594.s015.tif]

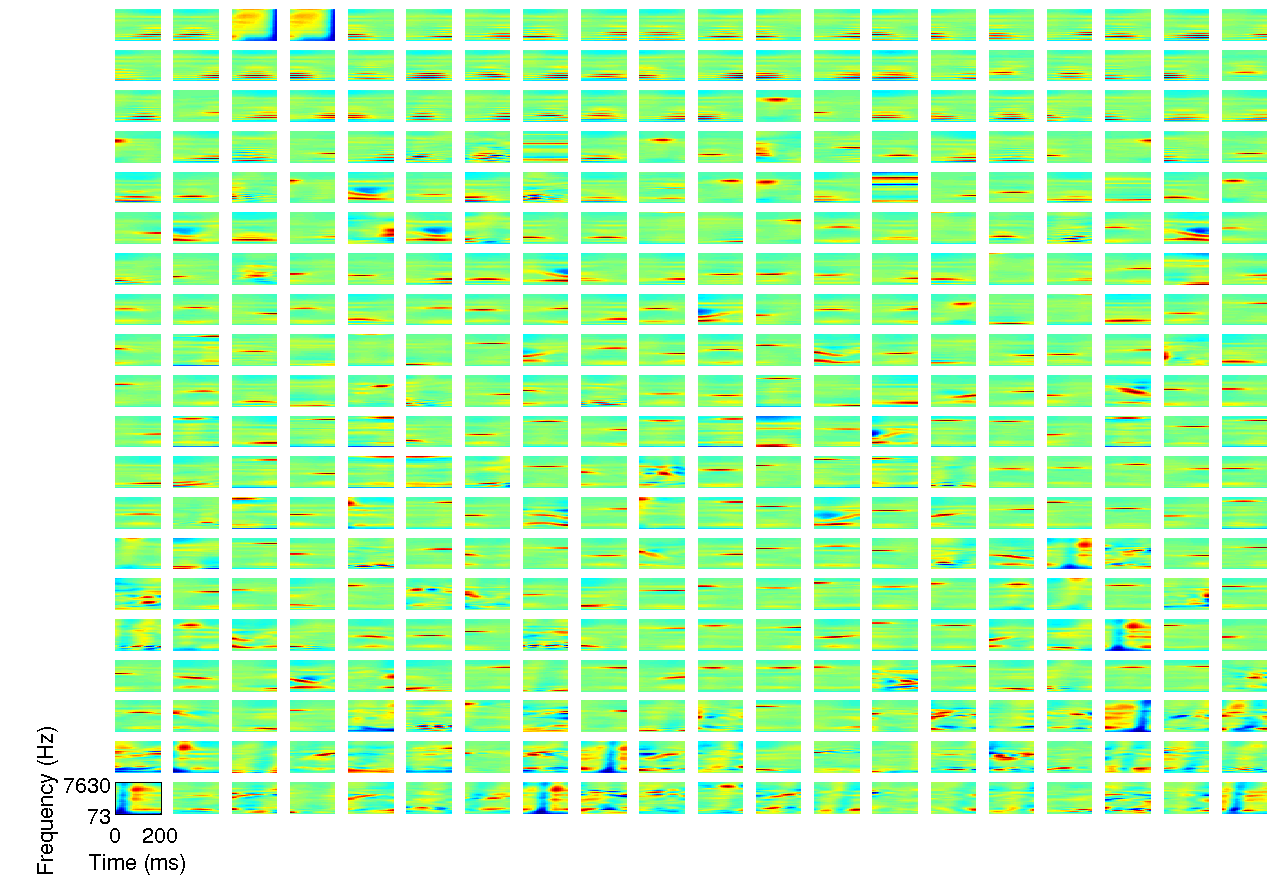

Supplement: Figure S16 — The full set of elements from a two times overcomplete, L1-sparse dictionary trained with LCA [24] on cochleograms of speech. Same conventions as Fig. S11. (TIF) [file pcbi.1002594.s016.tif]

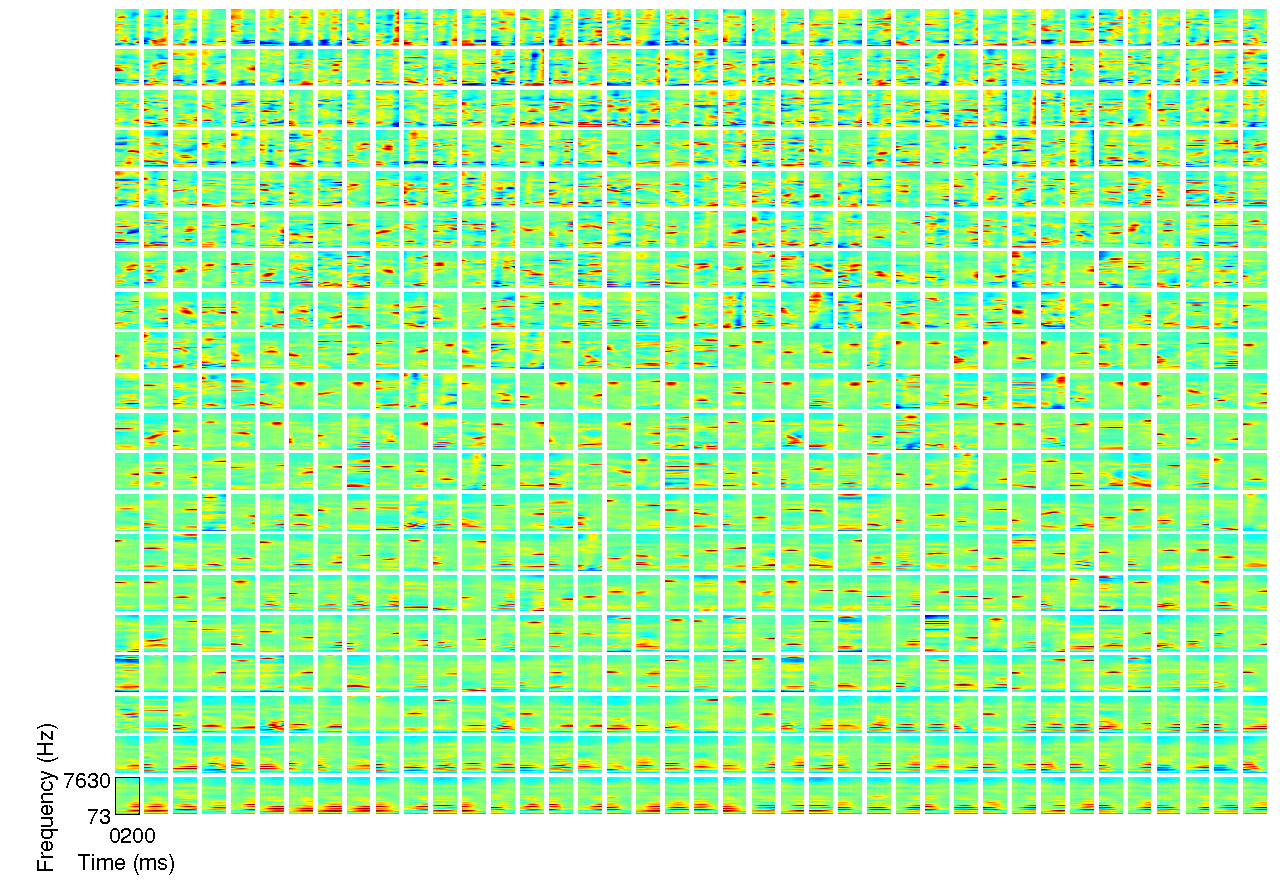

Supplement: Figure S17 — The full set of elements from a four times overcomplete, L1-sparse dictionary trained with LCA [24] on cochleograms of speech. Same conventions as Fig. S11. (TIF) [file pcbi.1002594.s017.tif]

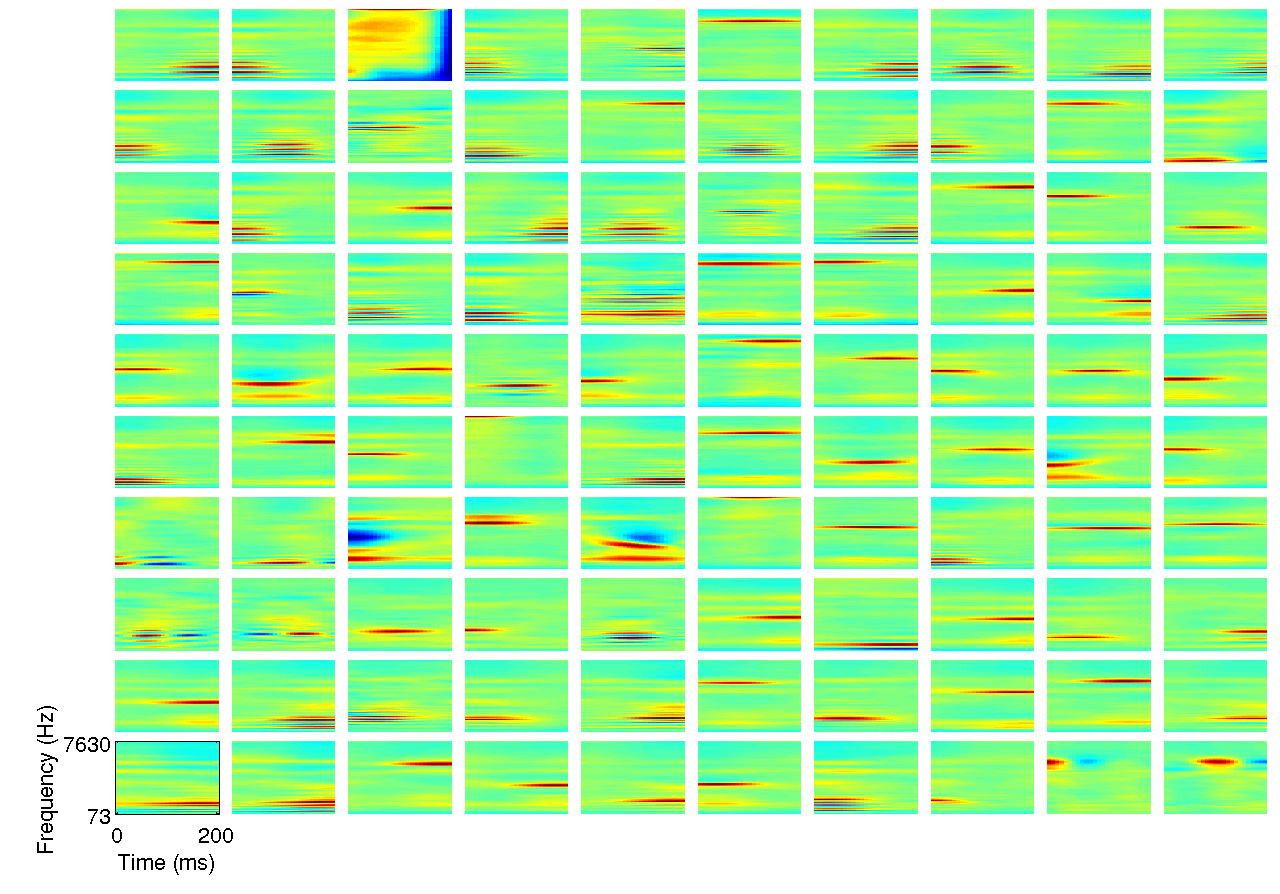

Supplement: Figure S18 — The full set of elements from a half-complete, L1-sparse dictionary trained with Sparsenet [10] on cochleograms of speech. Same conventions as Fig. S11. (TIF) [file pcbi.1002594.s018.tif]

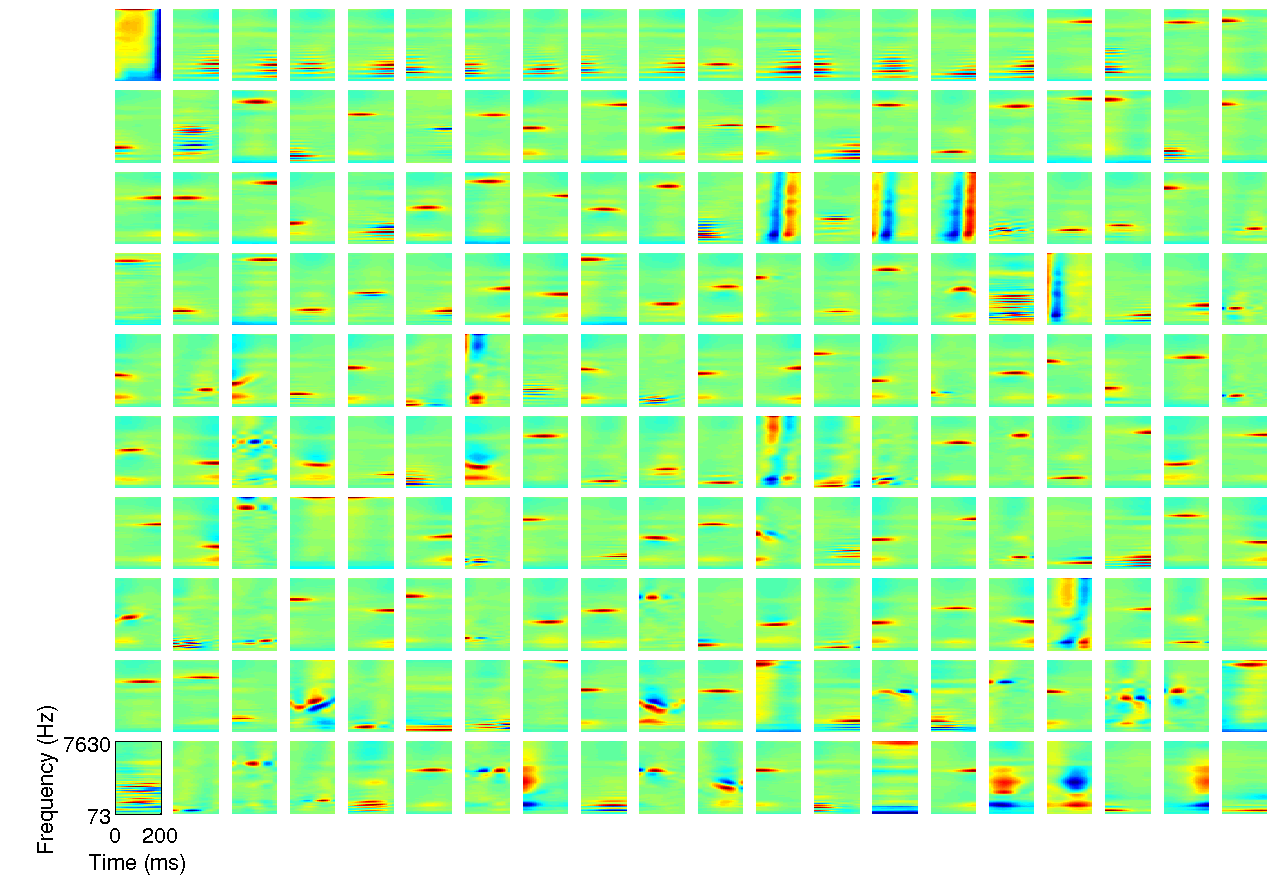

Supplement: Figure S19 — The full set of elements from a complete, L1-sparse dictionary trained with Sparsenet [10] on cochleograms of speech. Same conventions as Fig. S11. (TIF) [file pcbi.1002594.s019.tif]

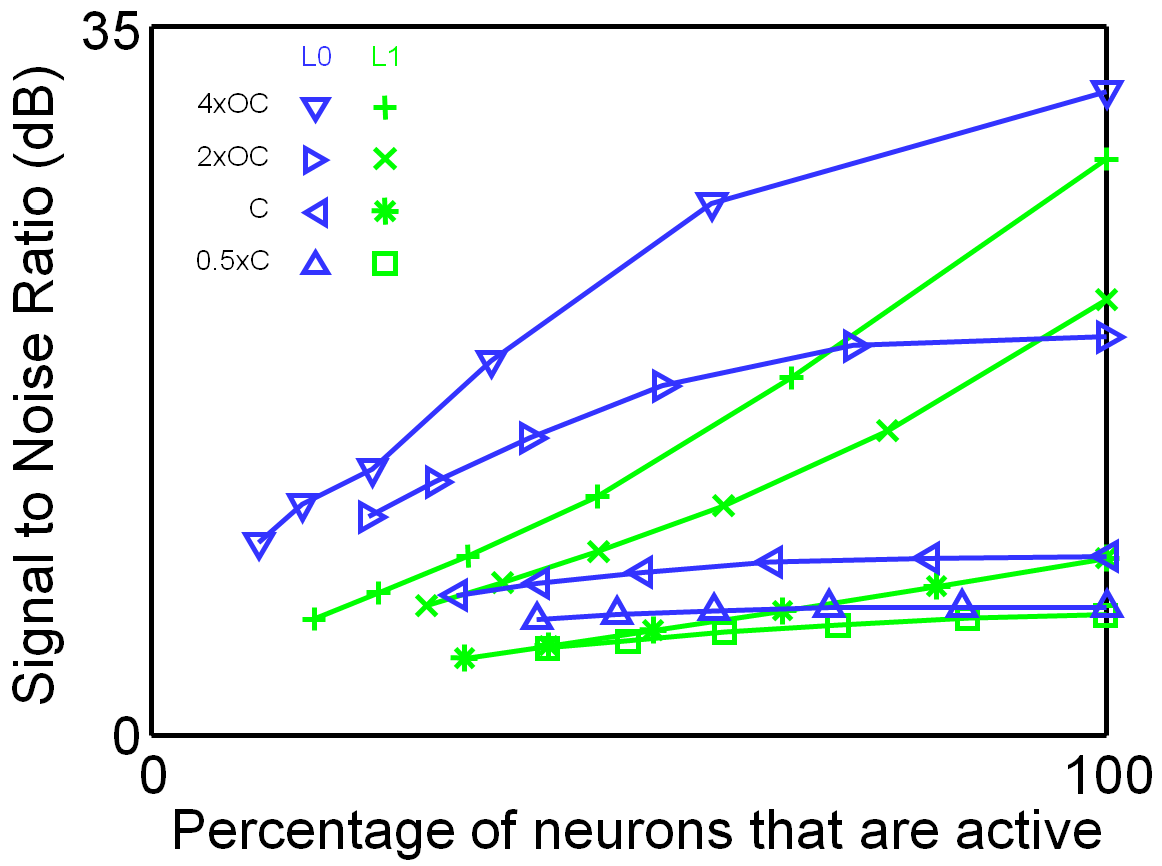

Supplement: Figure S20 — The signal to noise ratio (SNR) of sparse coding dictionaries increases with overcompleteness and with increasing numbers of active elements. Blue lines with triangles represent L0-sparse dictionaries, whereas green lines represent L1-sparse dictionaries. As expected, representations are more accurate with increasing numbers of active neurons and also when the level of overcompleteness is increased. Interestingly, the L0-sparse dictionaries typically have higher SNRs than the L1-sparse dictionaries. A few other general trends are evident as well. Most notably, the L0-sparse dictionaries have higher SNRs than the L1-sparse dictionaries for similar levels of sparseness. Also, the more overcomplete dictionaries have higher SNRs than half-complete ones, even with the same absolute number of active neurons. The half-complete and complete dictionaries do not show much improvement in performance even as the number of active neurons increases. Interestingly, we find that the performance of the L0-sparse dictionaries tend to saturate as the fraction of active neurons approaches unity whereas the corresponding curves for the L1-sparse dictionaries tend to curve upwards. Note that we did not optimize the dictionaries at each data point, but instead used the same parameters used when training the network. (TIF) [file pcbi.1002594.s020.tif]
